# Effectiveness of a Mobile-Based Influenza-Like Illness Surveillance System (FluMob) Among Health Care Workers: Longitudinal Study

**DOI:** 10.2196/19712

**Published:** 2020-12-07

**Authors:** May Oo Lwin, Jiahui Lu, Anita Sheldenkar, Chitra Panchapakesan, Yi-Roe Tan, Peiling Yap, Mark I Chen, Vincent TK Chow, Koh Cheng Thoon, Chee Fu Yung, Li Wei Ang, Brenda SP Ang

**Affiliations:** 1 Wee Kim Wee School of Communication and Information Nanyang Technological University Singapore Singapore; 2 School of New Media and Communication Tianjin University Tianjin China; 3 National Centre for Infectious Diseases Singapore Singapore; 4 The International Digital Health & Artificial Intelligence Research Collaborative (I-DAIR) Graduate Institute of Geneva Geneva Switzerland; 5 National University of Singapore Singapore Singapore; 6 KK Women’s and Children’s Hospital Singapore Singapore

**Keywords:** participatory surveillance, syndromic surveillance, mobile phone, influenza-like illness, health care workers

## Abstract

**Background:**

Existing studies have suggested that internet-based participatory surveillance systems are a valid sentinel for influenza-like illness (ILI) surveillance. However, there is limited scientific knowledge on the effectiveness of mobile-based ILI surveillance systems. Previous studies also adopted a passive surveillance approach and have not fully investigated the effectiveness of the systems and their determinants.

**Objective:**

The aim of this study was to assess the efficiency of a mobile-based surveillance system of ILI, termed FluMob, among health care workers using a targeted surveillance approach. Specifically, this study evaluated the effectiveness of the system for ILI surveillance pertaining to its participation engagement and surveillance power. In addition, we aimed to identify the factors that can moderate the effectiveness of the system.

**Methods:**

The FluMob system was launched in two large hospitals in Singapore from April 2016 to March 2018. A total of 690 clinical and nonclinical hospital staff participated in the study for 18 months and were prompted via app notifications to submit a survey listing 18 acute respiratory symptoms (eg, fever, cough, sore throat) on a weekly basis. There was a period of study disruption due to maintenance of the system and the end of the participation incentive between May and July of 2017.

**Results:**

On average, the individual submission rate was 41.4% (SD 24.3%), with a rate of 51.8% (SD 26.4%) before the study disruption and of 21.5% (SD 30.6%) after the disruption. Multivariable regression analysis showed that the adjusted individual submission rates were higher for participants who were older (<30 years, 31.4% vs 31-40 years, 40.2% [*P*<.001]; 41-50 years, 46.0% [*P*<.001]; >50 years, 39.9% [*P*=.01]), ethnic Chinese (Chinese, 44.4% vs non-Chinese, 34.7%; *P*<.001), and vaccinated against flu in the past year (vaccinated, 44.6% vs nonvaccinated, 34.4%; *P*<.001). In addition, the weekly ILI incidence was 1.07% on average. The Pearson correlation coefficient between ILI incidence estimated by FluMob and that reported by Singapore Ministry of Health was 0.04 (*P*=.75) with all data and was 0.38 (*P*=.006) including only data collected before the study disruption. Health care workers with higher risks of ILI and influenza such as women, non-Chinese, allied health staff, those who had children in their households, not vaccinated against influenza, and reported allergy demonstrated higher surveillance correlations.

**Conclusions:**

Mobile-based ILI surveillance systems among health care workers can be effective. However, proper operation of the mobile system without major disruptions is vital for the engagement of participants and the persistence of surveillance power. Moreover, the effectiveness of the mobile surveillance system can be moderated by participants’ characteristics, which highlights the importance of targeted disease surveillance that can reduce the cost of recruitment and engagement.

## Introduction

Seasonal influenza is an acute respiratory infection that causes an estimated 290,000-650,000 deaths worldwide [1]. As a major travel hub in a tropical area of southeast Asia, Singapore is at high risk of both imported southern and northern strains of seasonal influenza, exposing the country to the virus throughout the year without discrete peaks [2,3]. The economic burden of the influenza threat is estimated to produce 2.5 million lost days of work and US $470 million costs per year in Singapore [4,5].

Although there are existing influenza surveillance systems in Singapore, the timeliness and effectiveness of these systems can be improved. Since the influenza A (H1N1) outbreak in 2009, virological and clinical surveillance systems have been set up nationwide based on outpatients with influenza-like illness (ILI) visiting public health care institutions [2,6]. However, these methods can be less efficient due to the delay of visiting doctors or the culture of self-medication. This may especially be the case in Singapore because acute respiratory syndrome is often perceived as mild symptoms that can be self-cured or cured via alternative medicines such as traditional Chinese medicine [7-9]. In addition, despite the strength of existing surveillance systems, the maintenance cost can be high [10]. These limitations highlight the importance of a complementary surveillance system that is robust, in real-time, and cost-effective.

The recent idea of crowdsourcing has been promoting the evolution of participatory infectious disease surveillance, which has greatly improved the timeliness and effectiveness of influenza surveillance. Crowdsourcing in health surveillance encourages the general public to contribute their disease information via the internet. The United States, Australia, and Europe have been using internet-based influenza surveillance systems for over a decade [11-13]. The general public is invited via email to submit weekly reports about ILI and provide information on health care–seeking activities. The surveillance power of these internet-based systems is generally good. For example, ILI incidence estimated by Influenzanet (a European influenza surveillance system) in the Netherlands was found to have moderate to high correlations (ie, ranging from 0.42 to 0.89) with those estimated by traditional reporting systems during a 6-year period from 2003 to 2008 [14]. Similar findings were reported in other countries such as Belgium and Portugal using data collected across 10 years [13]. This suggests that internet-based participatory surveillance systems are a valid sentinel for ILI surveillance.

However, we recognize at least three significant limitations in extant work on participatory surveillance of influenza. First, current internet-based surveillance systems may have methodological limitations as they adopt a passive recruitment approach, for example via publicity campaigns involving television, radio, and newspaper, which engage several thousands of participants per season [13,14]. Although large samples can often yield better surveillance power, participant recruitment and system maintenance can be very costly and challenging using these methods [15]. Recruitment via mass advertisements has also been found to underrepresent important populations who are at high risk of ILI and influenza, such as children and the elderly [14]. Recent virological studies have demonstrated that targeted disease surveillance, which is based on specific populations such as university students and military recruits, is not only cost-effective but also complementary to existing systems [16,17]. However, the effectiveness of internet-based surveillance systems using targeted samples has not been thoroughly explored.

Second, although existing studies have demonstrated that current internet-based systems have good surveillance power [13], they have not examined how the power may vary between different populations from a targeted surveillance approach. It is reasonable to assume that people with different occupations, age groups, or lifestyles may demonstrate distinct effects on surveillance power. For example, the elderly, children, and those who have not been vaccinated against influenza are likely to be more vulnerable to the seasonal epidemic. These populations may be more suitable for the early detection of influenza epidemics. Therefore, it is important to understand what populations will demonstrate the highest surveillance power, which can strengthen the targeted surveillance approach and reduce recruitment costs.

Third, although previous studies also investigated determinants of participation in influenza surveillance [18], many studies have not considered determinants that are specific to mobile-based app systems, which are increasingly being used by the public and should be utilized. Unlike websites that can be rigid in their use, mobile apps can be utilized at any location within the user’s own timeframe [19]. This could assist in increasing participant engagement. However, mobile apps often require updates corresponding to the updating operating systems of mobile devices. Understanding these systematic pros and cons of different participatory approaches can highlight strategies to overcome the impact, increase engagement, and reduce maintenance costs.

To address the above limitations, the aim of this study was to present a mobile-based integrated surveillance system of ILI among health care workers, called FluMob, as a complementary system to the existing influenza surveillance in Singapore. FluMob is a digitally integrated syndromic surveillance system exclusively designed for health care workers in clinical settings (see detailed descriptions of the system in the Methods section) [20]. Health care workers are considered as a priority group for influenza preventive measures because they are at high risk of exposure to influenza. Health care workers account for 20%-30% of all influenza cases during regular influenza seasons worldwide [21,22]. In Singapore, this number can grow up to 40% in particular pandemics such as during the spread of severe acute respiratory syndrome (SARS) in 2003 [23]. In addition, health care workers can cause nosocomial outbreaks [24,25], because they can act as potential vectors for influenza transmission to high-risk patients. Lack of preventive measures of influenza for health care workers can lead to higher mortality and morbidity of older and immunocompromised patients [26,27]. Therefore, real-time surveillance systems targeted at health care workers are imperative to prevent health care costs due to additionally infected patients and the loss of workforce among health care workers. Such surveillance systems are invaluable as they can also be easily adapted to emerging respiratory infections such as COVID-19.

In this study, we tested the FluMob system over 2 years from 2016 to 2018 in health care worker populations. Data were collected on demographics, lifestyle, and weekly reports of ILI symptoms and health care–seeking behaviors. This study had two main objectives: (1) to evaluate the effectiveness of FluMob regarding the participation rate among health care workers and its surveillance power for ILI, and (2) to identify factors that can moderate the effectiveness of the app. The study was disrupted for maintenance of the system and the end of the incentive. Although we tried to minimize the potential impact of this disruption by notifying participants about the app relaunch, it is important to investigate whether and how these disruptions would affect the level of participation and surveillance power of the participatory surveillance system. This knowledge will offer invaluable lessons for future research. Thus, this study examined the following research questions: (1) What is the level of participation of FluMob among health care workers, and what factors would moderate the participation rate? (2) What is the level of ILI surveillance power of FluMob among health care workers, and what factors would moderate the ILI surveillance power?

## Methods

### Design and Setting

This study was approved by the National Health Group Domain Specific Review Board (DSRB Ref: 2014/01009) and the SingHealth Centralised Institutional Review Board. From April 2016 to March 2018, the research team administered the FluMob system in two large hospitals in Singapore: a designated hospital for screening and treatment during communicable disease outbreaks (Tan Tock Seng Hospital) and another that deals with pediatric patients (KK Women’s and Children’s Hospital). Between May 7 and July 15 in 2017, the system was shut down for maintenance. Participants were prompted with notifications of the app relaunch. More information on the development of the app and participant engagement can be found in Lwin et al [20].

### Intervention: FluMob System

FluMob is a digitally integrated syndromic surveillance system that integrates the ubiquitous access to the internet and the simple portability of smartphones. FluMob was developed using a mobile-based participatory epidemiological approach that relies on crowd involvement for disease surveillance and control through mobile technologies. Particularly, the system comprises a responsive web portal and mobile operating systems (ie, Android and iOS), allowing access from various types of mobile devices and web browsers. The FluMob app requires participants to register in the system upon their first use and to subsequently log in with a user identification and password. Health care workers can use the app easily and conveniently to provide reports of ILI on a weekly basis.

The system also has an analytic component for instantaneous surveillance. As soon as a report is submitted, the data input will be stored in a central database securely and confidentially. The research administrators (ie, the research team and other stakeholders) can access and view reports via an analytical module that is integrated into central servers. This allows researchers and clinicians to gather real-time surveillance of ILI, which can inform prevention and management strategies. A more detailed description of technical specifications and operating environments is available elsewhere [20].

### Participants and Procedures

A convenience sampling method was employed to recruit participants. Clinical and nonclinical hospital staff from all departments were invited via mass emails to participate in the study in April 2016. Respondents who were above 21 years old and owned mobile devices in Android or iOS systems qualified and were asked to download the FluMob app from the corresponding software store for free. A total of 200 health care workers from KK Women’s and Children’s Hospital and 500 health care workers from Tan Tock Seng Hospital were recruited and given a unique user ID in the system for data tracking.

Participants were informed that they would participate in the study for 18 months, although the study continued for a further 6 months. No notification was sent to participants when their official study end dates approached. Participants were provided 20 Singapore dollars (US $15) as an incentive if they submitted 10 or more reports for each half of the first year. To evaluate whether the participants would continue to use the app without the external motivator, we stopped the token of appreciation after 1 year of app use (ie, they were not paid after the first year). Notably, as the time of the system maintenance and that of the end of the incentive for most participants were well aligned, their effects could not be distinguished. Therefore, we termed this period as the “study disruption period” to indicate disruptions from both the system maintenance and the incentive end.

Upon registration, participants completed a background survey capturing demographics and influenza vaccination status. The data collected included age, gender, ethnicity, number of children in a household, job category and department in the hospital, and influenza vaccination records in the previous year. Eight participants did not complete the registration and thus were excluded from the sample. Two participants were excluded from the final sample because they did not submit a full background survey, with missing demographic and lifestyle information. Finally, 690 participants were included in the analysis.

On a weekly basis, participants were prompted via app notifications to complete a short questionnaire of acute respiratory symptoms through the FluMob app. They reported whether they had experienced any symptoms from a list since their last report, including fever, chills, runny or blocked nose, sneezing, cough, sore throat, shortness of breath, muscle or joint pain, headache, malaise, loss of appetite, colored phlegm, watery and bloodshot eyes, nausea, vomiting, diarrhea, stomach ache, and chest pain [28].

### Analysis Strategies

#### Individual Submission Rate and Weekly Reporting Rate

Although participants could submit weekly reports even after their study end dates, we excluded entries for estimations of individual participation rates and weekly submission rates. The individual submission rate was calculated at the individual level as the percentage of submitted weekly surveys over the number of weeks in the 18-month period excluding the period of system maintenance. The weekly reporting rate was calculated as the percentage of reporting participants over the cumulative number of participants at the week level.

To investigate the effect of the study disruption, we compared the individual submission rates before and after the disruption with a paired-sample *t* test. To understand the effectiveness of the implementation of FluMob in health care workers, we conducted a multivariable analysis, with the individual submission rate as the outcome and individual factors as predictors in a linear regression model. This analysis was designed to compare individual submission rates across participants by demographics, hospital departments, and vaccination status. To better understand the characteristics of participants who committed to submit weekly reports throughout the study, regardless of the study disruption, we defined participants who submitted at least two reports after the system maintenance as committed users. A multivariable logistic regression analysis was conducted using participant characteristics as predictors. All analyses were conducted with SPSS version 25.0 (IBM Corp, Armonk, NY, USA).

#### ILI Incidence and Surveillance Power

ILI was defined as fever (≥38.0°C) accompanied by a cough or a sore throat following the definition of the Ministry of Health (MOH) in Singapore [2]. As participants could submit weekly reports after their end dates of the study, we utilized all entries for the ILI incidence estimations. ILI incidence in week *i* was calculated by dividing the number of participants reporting ILI in week *i* by the total number of reports in week *i*.

On less than 1% of occasions in which participants submitted multiple surveys within a week, the survey that reported respiratory symptoms, or the latest report if none of the surveys reported symptoms, was retained in the analysis. If participants reported ILI symptoms in consecutive weeks, the symptoms were considered to be from the same episode [14,28]. Thus, the reporting of ILI in the later consecutive weeks was removed from the calculation of weekly ILI incidence.

To evaluate the surveillance power of the FluMob system, we compared the weekly ILI incidence based on FluMob (ILI%_FluMob) with the weekly ILI incidence reported by the Singapore MOH (ILI%_MOH) [29]. The ILI%_MOH was estimated as the ILI incidence among attendances for acute upper respiratory infection in the government-funded primary care clinics. The Pearson correlation coefficient between the 4-week moving proportion of ILI%_FluMob and the 4-week moving proportion of ILI%_MOH was used to measure the surveillance power of the FluMob system.

To examine the effect of the study disruption on the surveillance power of the FluMob system, we compared the surveillance correlation between ILI%_FluMob and ILI%_MOH estimated by all data with that estimated by only data collected before the disruption. Furthermore, to investigate what factors can determine the surveillance power of the system, we compared the surveillance correlations across different individual characteristics.

## Results

### Participant Demographics and Characteristics

**Figure 1:**
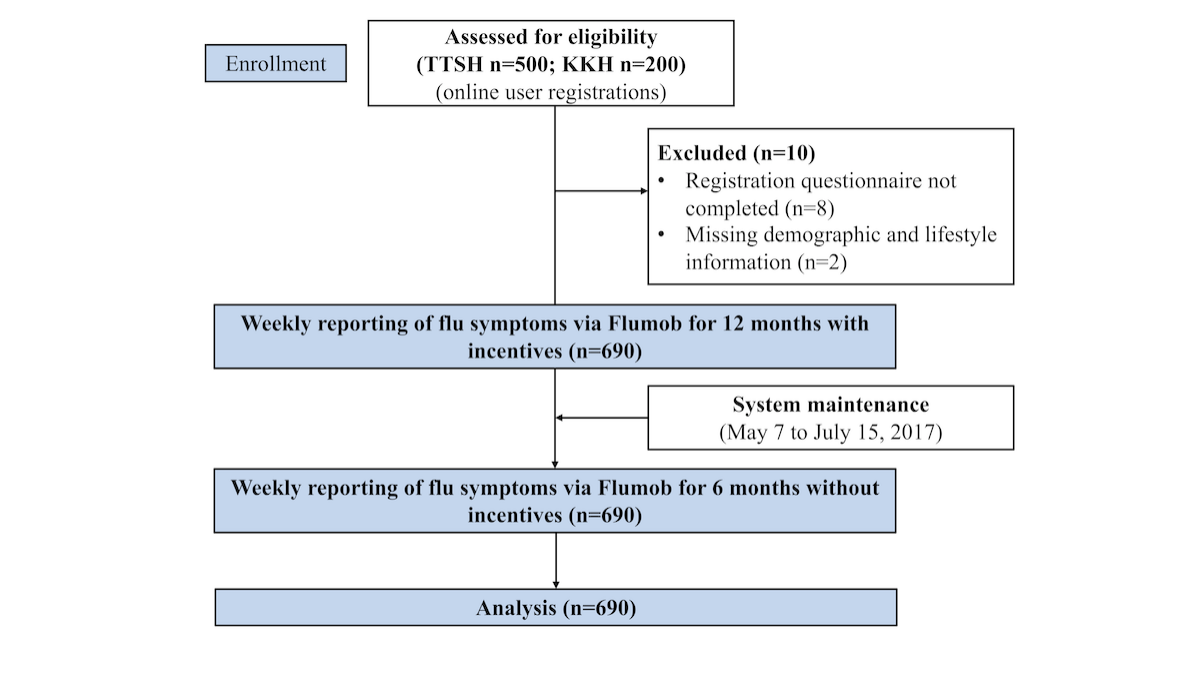
Participant flow chart. TTSH: Tan Tock Seng Hospital; KKH: KK Women’s and Children’s Hospital.

The participant flow diagram is shown in Figure 1. Table 1 shows the demographic and other characteristics of our sample. The majority of participants were women, aged under 40 years (531/690, 77.0%), and with no children in their households. Most of the participants reported receiving an influenza vaccination in the past year and had no allergic conditions.

**Table 1:** Demographic and other characteristics of the health care workers recruited for this study (N=690).

Characteristics		Participants, n (%)
**Gender**		
	Male	110 (15.9)
	Female	580 (84.1)
**Age (years)**		
	21-30	258 (37.4)
	31-40	273 (39.6)
	41-50	92 (13.3)
	>50	67 (9.7)
**Job category**		
	Administration and others	114 (16.5)
	Ancillary	105 (15.2)
	Allied health	186 (27.0)
	Medical	58 (8.4)
	Nursing	285 (41.3)
**Ethnicity**		
	Chinese	389 (56.4)
	Others	301 (43.6)
**Children in household**		
	Yes	127 (18.4)
	No	563 (81.6)
**Influenza vaccine in the past year**		
	Yes	564 (81.7)
	No	126 (18.3)
**Allergy**		
	Yes	178 (25.8)
	No	512 (74.2)

### Level of Participation

**Figure 2:**
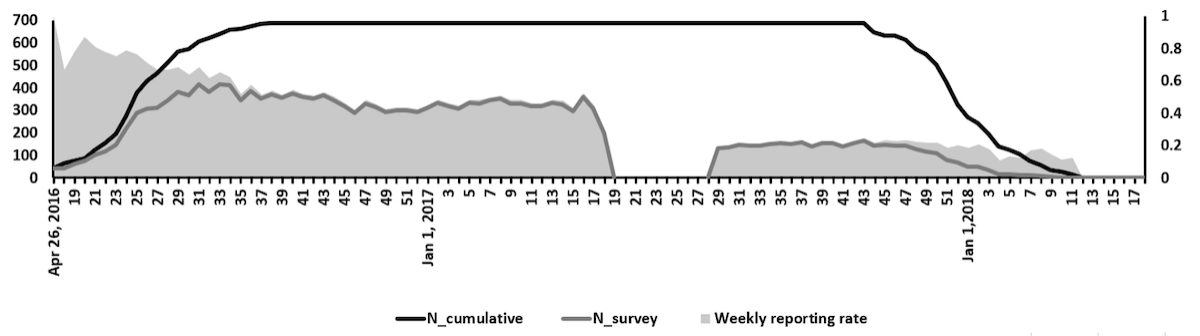
Weekly reporting rates among health care workers in FluMob. N_cumulative: cumulative sample size across time; N_survey: number of submitted surveys.

Among the 690 health care workers recruited for this study, 671 (97.2%) submitted at least one weekly symptoms survey after registration. The weekly reporting rate stabilized at about 50% before the study disruption but decreased to around 20% after the disruption (Figure 2). On average, the individual submission rate was 41.4% (SD 24.3%), with a rate of 51.8% (SD 26.4%) before the disruption and of 21.5% (SD 30.6%) after the disruption. The paired *t* test revealed that the average individual submission rate before the disruption was significantly higher than that after the disruption (t_689_=26.9, *P*<.001).

As shown in Table 2, the multivariate regression analysis indicated that participants aged below 30 years had lower individual submission rates than those aged above 30 years. Participants of Chinese ethnicity had a higher individual submission rate than participants of other ethnicities. Participants who had received an influenza vaccine in the year before the survey submitted more weekly reports than those who had not. No other significant results were found. In addition, to avoid the effect of the study disruption on the findings, we conducted the analysis for the period before the disruption, and similar results were observed (data not shown).

**Table 2:** Results of multivariable linear regression on individual submission rates by participant characteristics (N=690).

Characteristic			Adjusted submission rate		Unstandardized coefficient, estimate (SE)		*P* value
**Gender**							
	Female (Reference)	0.39		N/A^a^		N/A	
	Male	0.40		0.009 (0.026)		.74	
**Age (years)**							
	21-30 (Reference)	0.31		N/A		N/A	
	31-40	0.40		0.087 (0.021)		<.001	
	41-50	0.46		0.149 (0.029)		<.001	
	>50	0.40		0.084 (0.033)		.01	
**Job category**							
	Nursing (Reference)	0.39		N/A		N/A	
	Administration and others	0.42		0.030 (0.035)		.38	
	Ancillary	0.44		0.043 (0.027)		.12	
	Allied health	0.37		–0.019 (0.023)		.41	
	Medical	0.35		–0.045 (0.036)		.22	
**Ethnicity**							
	Chinese (Reference)	0.44		N/A		N/A	
	Others	0.35		–0.096 (0.019)		<.001	
**Children in household**							
	No (Reference)	0.40		N/A		N/A	
	Yes	0.38		–0.008 (0.024)		.74	
**Influenza vaccine in the past 1 year**							
	No (Reference)	0.34		N/A		N/A	
	Yes	0.45		0.102 (0.023)		<.001	
**Allergy**							
	No (Reference)	0.40		N/A		N/A	
	Yes	0.39		–0.002 (0.020)		.92	

^a^N/A: not applicable.

In total, 298 participants who submitted at least two reports after the system maintenance were identified as committed users. Multivariable logistic regression analysis (Table 3) showed that committed users were more likely to be above 30 years of age, ethnic Chinese, and those who were vaccinated in the previous year.

**Table 3:** Results of multivariable logistic regression of committed users by characteristics (N=298).

Characteristic		Committed users, n (%)	Adjusted odds ratio	95% CI	*P* value
**Gender**					
	Female (Reference)	252 (43.5)	1.00	N/A^a^	N/A
	Male	46 (41.8)	0.79	0.50-1.24	.31
**Age (years)**					
	21-30 (Reference)	95 (36.8)	1.00	N/A	N/A
	31-40	125 (45.8)	1.73	1.19-2.51	.004
	41-50	49 (53.3)	2.22	1.34-3.69	.002
	>50	29 (43.3)	1.36	0.77-2.40	.29
**Job category**					
	Nursing (Reference)	113 (39.7)	1.00	N/A	N/A
	Administration and others	31 (55.4)	1.79	0.98-3.26	.06
	Ancillary	53 (50.5)	1.48	0.95-2.42	.08
	Allied health	75 (40.3)	0.94	0.62-1.40	.74
	Medical	26 (44.8)	1.12	0.60-2.09	.71
**Ethnicity**					
	Chinese (Reference)	182 (47.0)	1.00	N/A	N/A
	Others	115 (38.2)	0.59	0.42-0.83	.002
**Children in household**					
	No (Reference)	248 (44.1)	1.00	N/A	N/A
	Yes	50 (39.4)	0.73	0.48-1.10	.13
**Flu vaccine in the past year**					
	No (Reference)	44 (34.9)	1.00	N/A	N/A
	Yes	254 (45.0)	1.59	1.05-2.40	.03
**Allergy**					
	No (Reference)	220 (43.0)	1.00	N/A	N/A
	Yes	78 (43.8)	1.04	0.73-1.48	.83

^a^N/A: not applicable.

### Level of ILI Surveillance Power

**Figure 3:**
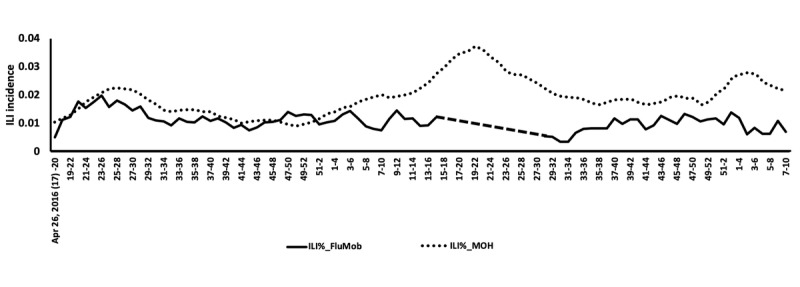
Comparisons between influenza-like illness (ILI) incidence estimated by FluMob (ILI%_FluMob) and that reported by the Singapore Ministry of Health (ILI%_MOH). Plotted values are 4-week moving proportions.

**Figure 4:**
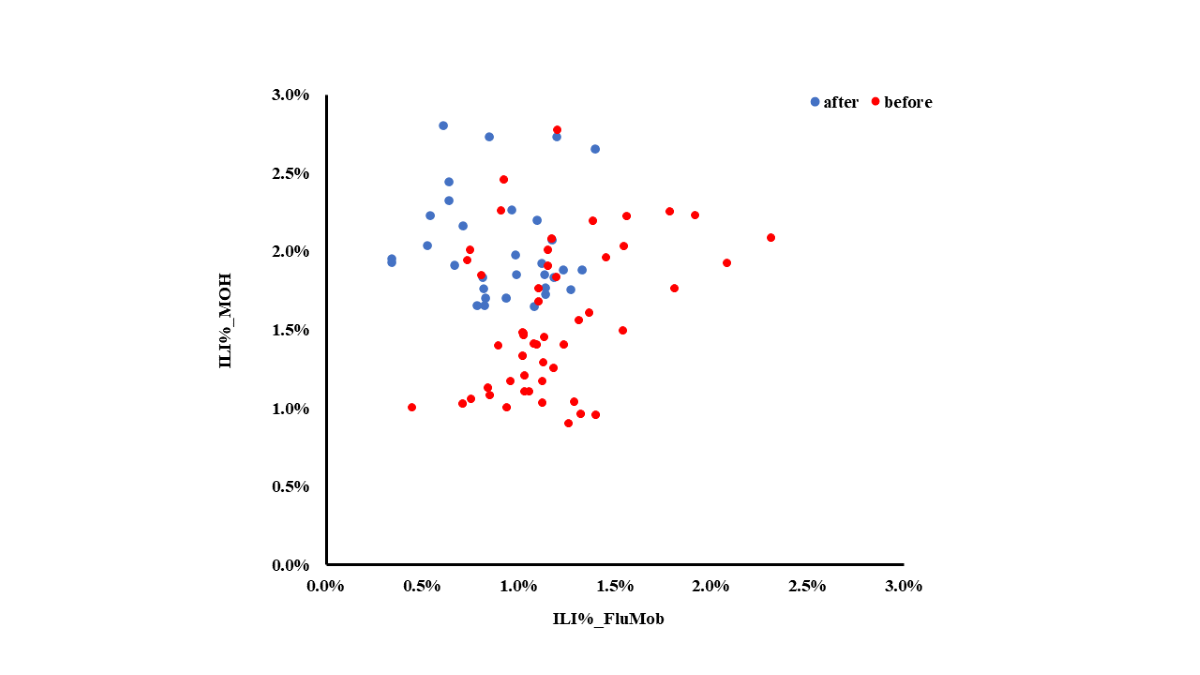
Scatter plot of percentage of influenza-like illness (ILI) estimated by FluMob and the Singapore Ministry of Health (MOH) using data collected before and after the system maintenance disruption.

Overall, 25.1% of all participants reported at least one episode of ILI during the investigation period. On average, the weekly ILI incidence was 1.07%. Figure 3 shows the 4-week moving proportions of ILI%_FluMob and ILI%_MOH. Figure 4 is a scatter plot of these two 4-week moving proportions before and after the system maintenance. Table 4 shows that when including all data, there was no significant linear relationship between the 4-week moving proportion of ILI%_FluMob and ILI%_MOH. However, when only data before the study disruption were used for analysis, the 4-week moving proportion of ILI%_FluMob was significantly correlated with that of ILI%_MOH.

Table 4 also shows the surveillance correlations by individual characteristics. With data before the study disruption, participants who were female, aged 41-50 years, non-Chinese, and with children in their households had higher surveillance correlations between ILI%_FluMob and ILI%_MOH. In addition, participants who worked in allied health, had not received an influenza vaccine in the past year, and reported allergy demonstrated higher surveillance correlations.

**Table 4:** Pearson correlations coefficients (r) between 4-week moving proportions of influenza-like illness incidence estimated by FluMob and those of Singapore Ministry of Health.

Variables			All data			Data before maintenance			
		*r*		*P* value		*r*		*P* value	
Overall		0.04		.75		0.38		.006	
**Gender**									
	Female	0.13		.25		0.46		.001	
	Male	–0.29		.007		–0.07		.62	
**Age (years)**									
	21-30	0.08		.47		0.31		.03	
	30-40	–0.16		.14		0.00		.99	
	41-50	0.35		.001		0.64		<.001	
	>50	–0.13		.24		–0.05		.73	
**Job category**									
	Administration and others	–0.11		.34		–0.23		.11	
	Ancillary	–0.16		.16		0.22		.12	
	Allied health	0.47		<.001		0.48		<.001	
	Medical	–0.21		.06		–0.19		.18	
	Nursing	–0.15		.17		0.19		.18	
**Ethnicity**									
	Chinese	–0.07		.53		0.10		.50	
	Others	0.12		.29		0.39		.004	
**Children in household**									
	Yes	0.12		.29		0.44		.001	
	No	–0.03		.79		0.25		.08	
**Influenza vaccine in the past year**									
	Yes	–0.14		.22		0.26		.06	
	No	0.52		<.001		0.45		.001	
**Allergy**									
	Yes	0.09		.42		0.44		.001	
	No	–0.03		.80		0.18		.20	

## Discussion

### Principal Findings

The primary aim of participatory disease surveillance systems is to provide a cost-effective and timely method for controlling and preventing epidemics. Internet-based platforms have enabled the gathering of disease information from the general public. Mobile-based apps can strengthen these surveillance platforms by integrating multifunctional and interactive components, and by accessing hard-to-reach populations. Targeted surveillance and knowledge of determinants of the system effectiveness are important, as they facilitate the planning and implementation of recruitment and reduce management costs. Toward this end, the aim of this study was to evaluate the effectiveness of a mobile-based ILI surveillance system for health care workers (ie, FluMob) and to examine the determinants of the effectiveness.

Our study suggests that mobile-based systems are feasible for respiratory infections surveillance within hospitals. The findings showed that health care workers were generally committed to participating in the ILI surveillance. They submitted one report every other week on average within the first year of surveillance, and approximately half of the participants regularly submitted weekly reports. Demonstrating this feasibility of targeted samples such as health care workers in hospitals is of practical significance, as it implies that our mobile-based system can be translated into regular surveillance to prevent outbreaks of influenza and other emerging respiratory infections in health care settings. Importantly, as the current COVID-19 outbreak has recurred after the initial pandemic wave, it is imperative to understand the effectiveness of our system to facilitate management of COVID-19 within the health care setting.

However, smooth operation of the system is essential to maintain its feasibility. In this study, only one-fifth of participants submitted weekly reports after the study disruption that included both a 10-week downtime of the system and the end of incentives. Unfortunately, we were not able to disentangle the effects of system maintenance and incentive end on participation due to the two events occurring consecutively. Nevertheless, these findings suggest that future research should consider the possible impacts of system maintenance and incentives on participation in surveillance programs.

The study findings highlight the potential of a targeted approach in participatory surveillance in several ways. First, our analyses show that individual characteristics are important determinants of participation. Greater participation was associated with older age and being vaccinated against influenza, which is consistent with past evidence suggesting that individuals with these characteristics are more concerned about their personal health and thus more likely to participate [12,18]. One novel finding was that being non-Chinese was associated with lower participation of ILI surveillance. This could be because of ethnic disparities in socioeconomic status and in turn health care–seeking behaviors in Singapore that favor the major Chinese group over the minority groups [30,31].

Second, these results demonstrate that mobile-based ILI surveillance of the targeted health care worker sample can be complementary to traditional surveillance based on the general public. The ILI incidence estimated by FluMob had a moderate correlation with that of the Singapore MOH within the first year of surveillance. However, the study disruption significantly weakened the surveillance correlation. We speculate that those who continued to submit weekly reports after the study disruption might be more health-conscious, which thus biased the ILI incidence estimations.

Third, and importantly, further analyses demonstrated that surveillance effectiveness could be distinct across different populations, thereby highlighting the potential of using a targeted sample for early detection of disease epidemics. Specifically, women had a higher power of ILI surveillance than men, possibly because women are more vulnerable to influenza due to physiological differences in sex [30,32]. Older age, not being vaccinated, and having allergic conditions were associated with higher surveillance power of ILI, which is likely because these are risk factors for influenza and other types of acute respiratory infections [28,33]. Thus, participants in those groups are more vulnerable to influenza and ILI epidemics. Furthermore, the ILI incidence estimated from health care workers who had children in their household demonstrated a higher predictive correlation with the ILI incidence reported by the MOH than those who did not, suggesting that they might be more exposed to illness via their young children [28]. Overall, the above findings suggest that demographic groups with higher risks of ILI and influenza would be more effective to target in ILI surveillance. This finding may be critical in future participatory surveillance research, as it facilitates the planning and implementation of sample recruitments using a targeted approach.

Future studies should include medical or laboratory confirmation of ILI to further boost accurate surveillance. However, and interestingly, the ILI incidence estimated by health care workers within FluMob was at a similar level to that reported by the MOH. This finding differs from previous studies showing that ILI incidence estimated by internet-based systems was often 3 to 10 times higher than that of the government reports [13,14,34]. One possible reason for this finding is that health care workers, especially those in the FluMob sample who work in hospitals for communicable disease and pediatric patients, are knowledgeable about acute respiratory symptoms.

Although our findings have significant theoretical and practical implications in ILI surveillance, the study has some limitations. The surveillance correlations found in this study (ie, *r*=0.0-0.4) were relatively low compared with those in previous internet-based surveillance studies [13,14]. Owing to the targeted approach, a smaller sample size was recruited in comparison to past research, which would inevitably lower the surveillance power. Furthermore, the system had a major disruption during the investigation. The downtime caused a loss of data during a big seasonal influenza outbreak in 2017, along with discouragement of participation and ultimately the loss of surveillance power. If such data were available, stronger surveillance correlations would have been obtained. Hence, such disruptions should be avoided with better study planning, funding support, and technological support. However, this disruption also offered us a valuable opportunity to investigate the impact of such breaks in accessibility on the effectiveness of mobile-based participatory ILI surveillance. Overall, this study was able to demonstrate a significant surveillance correlation, suggesting that the targeted surveillance approach can be an advantageous and complementary approach for participatory infectious disease surveillance.

### Conclusion

This study is among the first to evaluate the effectiveness of a mobile-based participatory ILI surveillance system and its determinants regarding participation engagement and surveillance power from a targeted health surveillance approach. The findings suggest that mobile-based systems can be effective for participatory surveillance. However, smooth operation of the mobile app without major disruptions is vital for the engagement of participants and the persistence of surveillance power. In addition, the effectiveness of the mobile-based system can be moderated by participants’ characteristics, which highlights the importance of targeted disease surveillance that can reduce the cost of recruitment and engagement. Our findings have significant theoretical and practical implications on participatory health surveillance based on mobile phones. Future research should identify factors that can improve the effectiveness of mobile-based participatory apps for infectious disease surveillance.

## Abbreviations

ILI: influenza-like illness
MOH: Ministry of Health
SARS: severe acute respiratory syndrome
